# Iliopsoas Pyomyositis With Bacteremia at an Early Stage of Presentation in a Temperate Region

**DOI:** 10.7759/cureus.26854

**Published:** 2022-07-14

**Authors:** Shiho Amano, Ryuichi Ohta, Chiaki Sano

**Affiliations:** 1 Community Care, Unnan City Hospital, Unnan, JPN; 2 Community Medicine Management, Shimane University Faculty of Medicine, Izumo, JPN

**Keywords:** japan, rural area, psoas sign, pyomyositis, psoas abscess

## Abstract

A psoas abscess, a collection of pus in the psoas muscle, is rare but the incidence is increasing with the use of computed tomography (CT) and magnetic resonance imaging (MRI). Pyomyositis, a muscular infection that does not lead to abscess formation, is well known as tropical pyomyositis because it is highly prevalent in tropical areas. We encountered a case of iliopsoas pyomyositis and bacteremia without abscess formation. The blood culture was positive despite the early stage of presentation and no abscess formation on MRI. Imaging is the gold standard for diagnosing iliopsoas abscesses. There are cases similar to ours wherein the blood culture is positive before the formation of an abscess. Regardless of whether an abscess is found on MRI or not, we need to consider the possibility of false negatives at the early stage of presentation. A suspicion of this disease is essential during a physical examination for early diagnosis and treatment, especially in rural areas, where medical resources are limited. Furthermore, pyomyositis is a common disease in tropical regions, but in recent years, case reports of occurrences in temperate regions have increased. This case indicates the need to consider pyomyositis as a differential diagnosis of fever and hip joint pain even in temperate regions.

## Introduction

A psoas abscess, a collection of pus in the psoas muscle, is rare. The previous report shows that its incidence rate is 0.4/100000, and the rate has been increasing with the use of computed tomography (CT) and magnetic resonance imaging (MRI) [1]. Psoas abscesses are categorized as primary or secondary based on their pathogenesis. A primary psoas abscess occurs as a result of the hematogenous or lymphatic transmission of infection from primary locations. The risk factors for primary psoas abscess include diabetes mellitus, HIV infection and other causes of immunosuppression, intravenous drug usage, and decreased renal function. A secondary psoas abscess occurs as a result of the direct spread of an adjacent infection into the psoas muscle [1].

The clinical findings of pyomyositis and psoas abscess may vary depending on the severity of the diseases at presentation. The symptoms of a psoas abscess include back or flank pain, fever, loss of appetite, malaise, weight loss, and an inguinal mass. Pain is present in 91% of cases and is localized in the back, flanks, and lower abdomen, but sometimes, it radiates to the hip and posterior thighs. Fever is present in 75% of cases [2-3]. The psoas sign, in which pain is induced by hip extension and stretching of the infected psoas muscle, is a practical physical examination finding. Due to the pain, the range of motion of the hip joint is limited, and patients frequently take a hip flexion and lumbar anteflexion position. The symptoms are nonspecific, and the onset is subacute, so the diagnosis may be challenging. A previous study showed a median time from onset to diagnosis of 22 days (more than 42 days in a third of patients) [3].

A psoas abscess is suspected based on symptoms and physical findings and diagnosed by imaging. Although CT is commonly used to detect psoas abscesses; its sensitivity may be low in the early stages. MRI is useful for detecting soft tissue and inflammation of vertebra [2-5]. Blood cultures are positive in 31.5% of cases. For primary and secondary psoas abscesses, the mortality rates are 2.4% and 19.0%, respectively, and 100% without treatment [2-3]. Delayed and inadequate treatment, aging, bacteremia, coronary artery disease, and Escherichia coli infection are risk factors for death [2].

Pyomyositis, a muscular infection that does not lead to abscess formation, is well known as tropical pyomyositis because it is highly prevalent in tropical areas. According to the International Statistical Classification of Diseases and Related Health Problems 11 (ICD-11), tropical pyomyositis occurs in the lower part of the psoas muscles (FB30) [2-3].

Pyomyositis is common in immunocompromised individuals (patients with either HIV, hematological disorders, or malignancies) in the tropical regions; however, it can also occur in healthy individuals after blunt trauma or as a secondary infection due to bacteremia. With recent globalization, it has also been observed in temperate regions [6-7]. Pyomyositis often develops in the large muscles of the extremities, particularly in the muscles of the lower extremities, including the iliopsoas, quadriceps, and gluteus maximus muscles [6-7].

We encountered a case of iliopsoas myositis and bacteremia without abscess formation. In this case, despite the early stage of presentation, the blood culture was positive, and no abscess formation was observed on MRI. Iliopsoas pyomyositis is less common in Japan, a temperate region; thus, there is a high possibility that its diagnosis may be difficult and delayed. This case did not involve any obvious immunodeficiency and occurred in a temperate region, and iliopsoas pyomyositis needs to be considered as a differential diagnosis of back or flank pain with fever even in temperate regions.

## Case presentation

A 79-year-old woman with full activities of daily living (ADL) was admitted to a rural community hospital with acute-onset right hip pain and fever. She was able to walk until the day before admission, but thereafter, she had difficulty moving due to pain caused by the extension of her right hip joint. Her medical history included chronic subdural hematoma, Alzheimer’s dementia, and Sjögren syndrome. Her medications included mecobalamin 1500 mcg, kallidinogenase 75 unit, Goreisan (Japanese Kampo) 7.5 g, eldecalcitol 0.75 mcg, Limaprost alfadex 15 mcg, donepezil 5 mg, pravastatin sodium 5 mg, and ramelteon 8 mg.

The vital signs at the time of admission were as follows: blood pressure of 124/75 mmHg, heart rate of 78 beats per minute, SpO2 of 98%, respiratory rate of 18 beats per minute, and body temperature of 38.2°C. The patient's right psoas sign was positive. There was no rash or swelling in the legs. A systolic murmur was heard but there were no apparent crackles and wheezes on the chest. She had multiple scratches on her lower legs. There were no other abnormalities in physical examinations. Initial laboratory tests revealed elevated neutrophil count, but no pyuria was observed (Table 1).

**Table 1:** Initial laboratory tests on admission day PT-INR, prothrombin time-international normalized ratio; APTT, activated partial thromboplastin time; SARS-COV-2, severe acute respiratory syndrome coronavirus 2

Marker	level	Reference
White blood cells	13.0 ×10^3^	3.0-7.8×10^3 ^/μL
Neutrophils	85.0	40-69 %
Lymphocytes	6.7	26-46 %
Monocytes	8.1	3-9 %
Eosinophils	0.0	0-5 %
Basophils	0.2	0-2 %
Red blood cells	403 ×10^4^	353-466 ×10^4 ^/μL
Hemoglobin	13.2	10.6-14.4 g/dL
Hematocrit	40.0	32.1-42.7 %
Mean corpuscular volume	99.1	83.3-100.3 fl
Platelets	21.9	13.8 /μL
PT-INR	0.94	0.85-1.15
APTT	28.8	24-39 second
D-dimer	5.3	<1.0 μg/ml
Blood urea nitrogen	13.3	7-24 mg/dL
Creatinine	0.46	0.4-1.1mg/dL
Sodium	138	135-147 mEq/L
Potassium	3.5	3.3-4.8 mEq/L
Chloride	101	98-108 mEq/L
Calcium	9.7	8.6-10.4 mg/dL
Phosphorus	2.9	2.5-4.5 mg/dL
Total protein	7.7	6.5-8.0 g/dL
Albumin	4.2	4.0-5.2 g/dL
Total bilirubin	1.1	0.2-1.2 mg/dL
Aspartate transaminase	20	8-38 IU/L
Alanine transaminase	19	4-43 IU/L
Alkaline phosphatase	101	38-113 U/L
gamma-glutamyl transpeptidase	12	<48 IU/L
blood sugar	144	78-109 mg/dL
SARS-COV-2 antigen	(-)	
Urine test		
leucocyte	(-)	
nitrite	(-)	
protein	(-)	
glucose	(+-)	
urobilinogen	normal	
bilirubin	(-)	
ketone	(1+)	
blood	(-)	
pH	8.0	
specific gravity	1.016	

**Figure 1:**
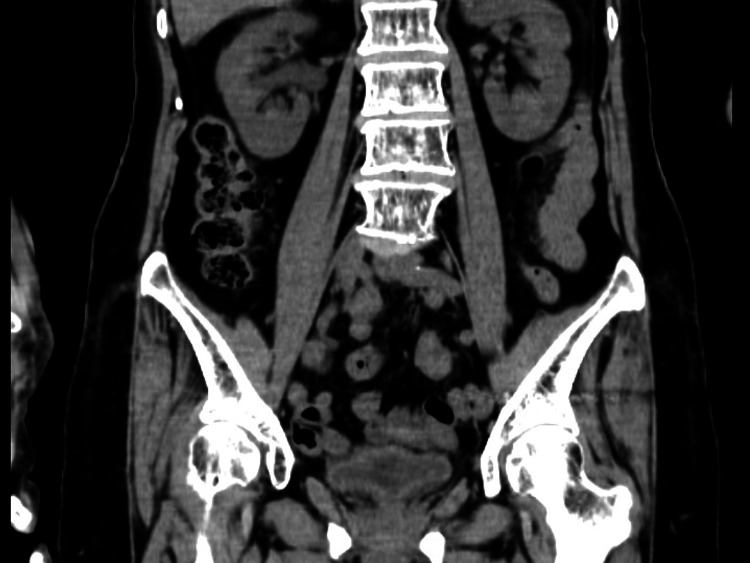
Abdominal computed tomography (coronal): showing no obvious abscess formation or fracture findings

The plain abdominal CT performed to identify the source of fever and pain showed no obvious abscess formation or fracture (Figure 1).

The same day, she was admitted for further examination and treatment.

**Figure 2:**
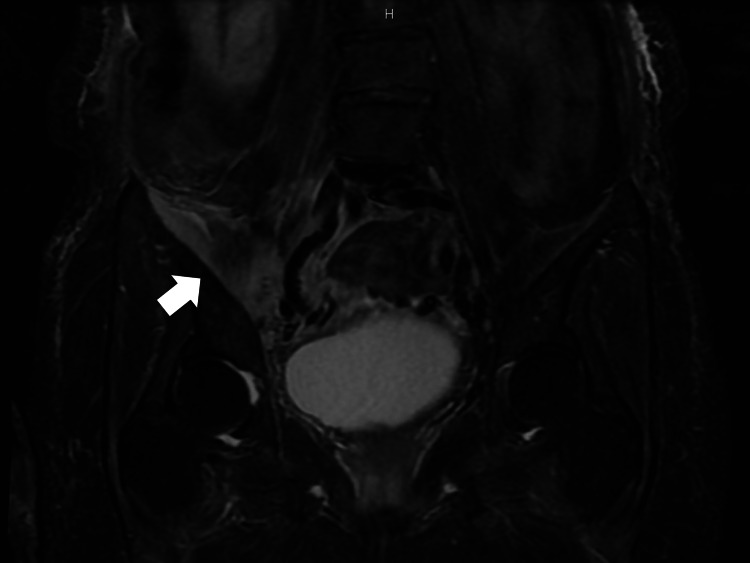
Magnetic resonance imaging (MRI, short-TI inversion recovery (STIR)) of the lumbar and pelvic: showing inflammation of the iliopsoas muscle (white arrow)

On the second day of hospitalization, gram-positive cocci grew from the blood culture. Treatment with cefazolin (CEZ) (4 g/day) was initiated, considering the possibility of Staphylococcus aureus infection. On the third day of hospitalization, lumbar and pelvic MRI revealed inflammation of the iliopsoas muscle (Figure 2).

Abscess formation was not observed. There were no findings of infection in the vertebral bodies or intervertebral discs. Based on the above blood culture results, the patient was diagnosed with methicillin-sensitive *Staphylococcus aureus *(MSSA) bacteremia due to iliopsoas pyomyositis.

Considering the possibility of infective endocarditis, transthoracic echocardiography was performed, but there was no vegetation. On the fifth day of hospitalization, the blood culture result confirmed MSSA; therefore, intravenous CEZ was continued for 14 days after the confirmation of negative blood culture. The oral cavity was not considered an entry site for MSSA bacteremia. We considered that the cause of bacteremia was an invasion of the skin of the lower leg because of poor hygiene. After commencing CEZ, the pain in the right hip gradually improved. The patient was discharged from the hospital after switching medication to the oral form of 2 g/day for two weeks. Her ADL improved to the same level as previously.

## Discussion

This case described blood culture-positive iliopsoas pyomyositis before abscess formation. Although a CT scan or MRI is required for the diagnosis of iliopsoas abscesses, sensitivity and specificity are low in the early stages of the infection [4]. A previous study showed that the sensitivities of plain CT, contrast-enhanced CT, and plain MRI were 100% six days after onset, whereas within five days, they were 33%, 50%, and 50%, respectively [5]. In this case, MRI was performed on the third day of onset, detecting no abscess. Considering that it was examined earlier, it is possible that the condition was pyomyositis before abscess formation. Similar to our case, there are cases in which the blood culture is positive even before the formation of an abscess, and even if no abscess is found on MRI, the possibility of false-negative results in the early stage of the presentation should be considered.

Pyomyositis clinically presents in three stages: (1) invasive stage: during which dull pain is accompanied by low-grade fever and malaise, with swelling appearing over one to three weeks; (2) suppurative stage: marked by tenderness, swelling, and severe myalgia appearing over 10-21 days; and (3) the late stages: with sepsis and marked pain [8]. In our case, admission occurred immediately after onset, at the invasive stage, considering the onset period. However, the blood culture was shown to be positive at this time, although the progression of pyomyositis takes time. Pyomyositis is often reported in tropical regions, and in Japan, it is rarely considered a differential diagnosis compared to an iliopsoas abscess. Considering the rate of progression of iliopsoas abscesses and the time to diagnosis, the condition that is usually diagnosed as an iliopsoas abscess may be pyomyositis.

The positive blood culture rate for pyomyositis is 5-35%, which tends to be lower than that for iliopsoas abscesses [8]. In our case, the physical findings showed a positive psoas sign and the blood culture was positive; however, no abscess was found on MRI. Therefore, the patient was diagnosed with iliopsoas pyomyositis. Delayed treatment and positive blood culture are risk factors for death; therefore, accurate diagnosis and early initiation of treatment are required.

Imaging is the gold standard for diagnosing iliopsoas abscesses. Since a psoas sign can be recognized at the time of admission, as in this case, this suggests the possibility that early diagnosis and treatment can be based on physical findings. In rural temperate regions, available medical resources are limited. In particular, it is difficult to perform MRI examinations during holidays and after hours. Given the false-negative MRI results at the early stage of presentation, suspicion of disease based on physical findings and laboratory data is required to initiate early treatment. The most common bacterial organism is S. aureus, which includes methicillin-resistant SA (MRSA) in 62-90% of cases [6,8-10]. Other organisms include *Streptococcus pyogenes, Salmonella, Escherichia coli, *and *Pneumococcus*. MRSA has been reported to be present in approximately 25-30% of all *S. aureus *isolates [9-10]. In cases with poor responsiveness to cefazolin or in areas where MRSA is frequently detected, antibiotics are required as first-line treatment against *S. aureus*, including MRSA infection. Additionally, gram-negative bacilli may be the causative organism in immunocompromised patients and may require the use of broad-spectrum antibiotics such as vancomycin and carbapenem.

Furthermore, in older people, the symptoms could be vague; therefore, physicians should consider history-taking and physical examinations specific to rural contexts, including many older people. Older people’s help-seeking behaviors, such as self-management and medical care usage, could be associated with their backgrounds [11]. Rural older adults tend to avoid revealing their symptoms explicitly because they prefer to conceal their weaknesses [12]. In medical facilities, elderly people usually visit health facilities with their families and are then reluctant to show their symptoms [13]. Their hesitancy may delay the diagnosis of critical diseases such as abscesses in hidden parts of the body. Rural physicians should consider this tendency in older people and ensure that they obtain detailed medical histories and perform thorough physical examinations.

## Conclusions

We encountered a case of iliopsoas pyomyositis with a blood culture-positive result and no abscess formation. Although imaging is the gold standard for diagnosing this disease, disease suspicion is necessary based on the physical examination to facilitate early diagnosis and treatment, especially in rural areas where medical resources are limited. Although pyomyositis is a common disease in tropical regions, case reports in temperate regions have increased in recent years. This indicates the need to consider pyomyositis as a differential diagnosis even in temperate regions.
